# Understanding and Targeting Natural Killer Cell-Cancer-Associated Fibroblast Interactions in Pancreatic Ductal Adenocarcinoma

**DOI:** 10.3390/cancers13030405

**Published:** 2021-01-22

**Authors:** Zoe X. Malchiodi, Louis M. Weiner

**Affiliations:** Department of Oncology, Georgetown Lombardi Comprehensive Cancer Center, Georgetown University Medical Center, 3870 Reservoir Road NW, Washington, DC 20057, USA; weinerl@georgetown.edu

**Keywords:** natural killer (NK) cells, cancer-associated fibroblasts (CAFs), pancreatic stellate cells (PSCs), pancreatic ductal adenocarcinoma (PDAC), tumor microenvironment (TME), fibroblast activation protein (FAP), alpha smooth muscle actin (αSMA)

## Abstract

**Simple Summary:**

Pancreatic cancer is an aggressive disease with a 5-year survival rate of less than 10%. Current therapies can be ineffective due to immune suppression and fibrosis (tissue scarring) that prevents cancer cells from being killed. This review article discusses the relevance of examining how natural killer (NK) cells, immune cells involved in the anti-cancer immune response, interact with cancer-associated fibroblasts (CAFs), which cause fibrosis, in pancreatic cancer. Understanding how these cell types interact may provide insights to guide the development of novel targeted therapies to increase immune response and survival in patients with pancreatic cancer.

**Abstract:**

Interactions between natural killer (NK) cells and cancer-associated fibroblasts (CAFs) comprise a relevant but relatively understudied crosstalk relationship within the tumor microenvironment (TME). This review discusses the relevance of both natural killer cell and cancer-associated fibroblast function and activity in cancers, with an emphasis on pancreatic ductal adenocarcinoma (PDAC), incorporating additional insights from other malignancies to inform future directions for research. We describe what is currently known about NK cell-CAF crosstalk and their molecular interactions, how it is possible to exploit NK cell cytotoxicity in tumors and how to target CAFs to enhance efficacy of cancer therapies and cytotoxic immune cells. Although not previously tested in combination, there is an abundance of evidence demonstrating that targeting tumor-promoting CAFs and exploiting NK cells, separately, are beneficial as therapeutic strategies. This raises the possibility that a novel combination regimen addressing these two cell targets may be even more beneficial to eradicate PDAC and other solid tumors.

## 1. Introduction

The TME is a mixture of cancer cells, fibroblasts, extracellular matrix (ECM) proteins, endothelial cells, and immune cells, including myeloid derived suppressor cells (MDSCs), macrophages, neutrophils, and antigen-presenting dendritic cells (DCs); T-cells and natural killer cells. All of these cellular components interact to mediate or inhibit tumor progression, but relationships between NK cells and CAFs have not been well studied.

NK cells are cytotoxic immune cells of the innate immune system. Unlike analogous members of the adaptive immune system, NK cells are known to lyse target cells, like cancer cells, without prior sensitization to the target cell [1]. Complex activating and inhibitory signaling responses in NK cells mediate their function. Understanding this complex biology has allowed NK cell cytotoxicity to be exploited in cancer therapies, like utilizing cytokine therapies, chimeric antigen receptor (CAR)-NK cell therapy, immune checkpoint inhibitors (ICIs) for CTLA-4 (cytotoxic T-lymphocyte-associated protein-4), PD-1 (CD279) and PD-L1 (CD274), and the use of inhibitors of inhibitory NK cell receptor-ligand interactions. Interestingly, there is novel evidence that NK cells can also target and interact with cancer-associated fibroblasts.

CAFs are heterogeneous fibroblast populations that greatly influence immune cell activity and function within the TME, mostly promoting immune suppression via secretion of cytokines and chemokines to inhibit cytotoxic immune cells or to induce proliferation of inhibitory immune cell populations [2]. A higher abundance of CAFs and CAF markers is correlated with increased tumor fibrosis and worse overall survival in patients [3,4,5]. Two distinct subpopulations of CAFs are known: myofibroblasts and inflammatory fibroblasts that have distinct phenotypes and localizations in the stromal compartment but contain variable expression of proteins present in both subpopulations, like fibroblast activation protein (FAP) [4,5,6,7]. Previous experiments targeting CAFs in vivo illustrated that alpha smooth muscle actin (αSMA)-positive CAFs, primarily myofibroblastic, and Sonic Hedgehog (Shh)-positive CAFs are both tumor restrictive, while targeting FAP^+^ CAFs unveiled a targetable tumor-promoting CAF subpopulation and has clinical promise. CAFs have also become ideal targets because they are less likely to develop resistance to cancer therapies [8]. However, CAFs impose therapeutic challenges by exhibiting plasticity [3,4,5,6,7,8,9], illustrating the need to further understand the function of CAF subpopulations in order to develop improved targeted therapies against tumor-promoting CAFs.

A combination of depleting CAFs and enhancing NK cell activity may increase immune cell infiltration into tumors by overcoming immune suppressive tumor-promoting CAFs, and by decreasing fibrosis, which may be a novel therapeutic strategy for highly fibrotic tumors. There are many examples of separately depleting or targeting CAFs and enhancing NK cell cytotoxicity both preclinically and clinically, but not in combination. Therefore, the aim of this review is to contextualize available knowledge about these NK cell-CAF interactions, in the hope of stimulating further research in this area.

## 2. NK Cell Function and Activity in Normal Biology and Malignancies

NK cells exist as heterogenous populations. The two main subtypes in humans are CD56^bright^CD16^dim^ and CD56^dim^CD16^bright^, with the latter being mature NK cells and generally exhibiting more cytotoxicity against its targets than the former [1,10]. CD56 is a known marker for human NK cells. CD16 is a marker of NK cell activation and maturation and is a FcγRIII receptor [1], which is a type of Fc receptor with low affinity for aggregated immunoglobulin-G (IgG) molecules [8], which is critical for mediating antibody-dependent cellular cytotoxicity (ADCC) by NK cells [11,12,13,14]. Other important NK cell markers include CD27 and CD107α (LAMP-1), which are markers of NK cell activation and degranulation, respectively [11]. Degranulation occurs when NK cells become activated and release perforin and granzyme B towards a target cell. Perforin is a protein that creates pores in the plasma membrane of target cells, while granzyme B can enter the pores to cleave and activate caspases within target cells to initiate apoptosis. NK cells also express tumor necrosis factor (TNF) superfamily proteins like Fas ligand (FasL) and TRAIL (TNF-related apoptosis-inducing ligand) to induce the extrinsic apoptosis pathway in a contact dependent manner [15,16] with target cells.

NK cells function by secreting inflammatory cytokines and chemokines in a contact dependent manner [17,18] with their target cell. Key cytokines produced by NK cells include interferon-γ (IFN-γ) and TNF-α, while important chemokines secreted by NK cells are CCL1-5 and CCL8 [15], which are necessary for NK cell migration. IFN-γ is one of the most abundant cytokines secreted by NK cells and is known to activate antitumor immunity and is involved with expression of components in both the intrinsic and extrinsic apoptotic pathways. However, exogenous IFN-γ reduces the expression of activating receptors on NK cells, like NKG2D [15], providing a feedback mechanism for NK cells. TNFα is a pro-inflammatory cytokine that can also increase INF-γ secretion by NK cells [15].

NK cells express both activating and inhibitory receptors on the cell surface and engage ligands on the cell surface of target cells. Mature NK cells usually co-express both activating and inhibitory receptors to prevent autoreactivity [19]. Different types of NK cell receptors include natural cytotoxicity receptors (NCRs), killer-IgG-like receptors (KIRs), the C-type lectin-like family of receptors (Ly49s) in mice and signaling lymphocytic activation molecules (SLAMs). SLAMs are expressed on all immune cells but play a role in NK cell activation [20]. NKp30, NKp44, and NKp46 are all type I immunoglobulin (Ig) transmembrane receptors where NKp30 and NKp44 can also be expressed on T-cells, primarily γδT-cells in different tumor models but at significantly lower levels [21,22]. NKG2D is a C-type lectin-like receptor and is in the type II CD94 transmembrane receptor family [15]. DNAM-1 (DNAX accessory molecule-1; CD226) is an Ig superfamily molecule involved with NK and T-cell-mediated cytotoxicity [20,23]. KIRs are also Ig superfamily receptors that recognize major histocompatibility complex (MHC) I molecules, particularly human leukocyte antigen (HLA) class I molecules [24]. NCRs and KIRs are present in humans, while Ly49s are KIR isoforms expressed in mice. Table 1 includes a list of known NK cell receptors and ligands, describing their impacts on activation or inhibitory states in both human and murine isoforms.

NK cell activating receptors can recognize targets on transformed, stressed, or activated cell populations from both primary tumors and metastases [15,33,34,35]. Interestingly, NCRs play an important role in regulating metastasis. In a *Ncr1* KO murine model, there was increased metastasis caused by decreased Ncr1-regulated production of both TNFα and IFN-γ, the latter also directly decreased the ECM protein fibronectin 1 (FN1) in both melanoma and lung adenocarcinoma models [25,35]. Decreased expression of IFN-γ and FN1 compromised the TME architecture to a more “aggressive” phenotype mediating metastasis in these models [25,35]. Additionally, NK cell depletion increased metastasis [25,35] suggesting that NK cells and expression of their activating receptors are necessary to prevent metastasis and tumor progression. However, non-malignant target cells express MHC I molecules, which are recognized by NK cell inhibitory receptors. This co-expression of activating and inhibitory receptors, and MHC I downregulation are processes by which NK cells employ self-recognition to avoid an autoimmune response. Unlike normal cells, cancer cells are known to downregulate MHC I molecules, signaling NK cells to bind to them due to lowered expression of inhibitory NK cell ligands and increased expression of NK cell activating ligands on cancer cells, making them potentially sensitive to lysis by NK cells [15]. Despite lower MHC I presentation, cancer cells are resistant to NK cell mediated lysis, which can be caused by multiple mechanisms, including: (1) increasing expression of NK cell inhibitory ligands, (2) secreting tumor growth factor β (TGFβ) to prevent NK cell activation, (3) secreting soluble NK cell ligands into the ECM to prevent NK cell contact with the tumor cell, a process that may be mediated by metalloproteinases (MMPs) from both cancer cells and CAFs [36,37,38,39]; or (4) interact with other cellular components of the TME, like CAFs [12,36].

## 3. Cancer-Associated Fibroblasts

In normal tissue, fibroblasts are generally quiescent, or in a resting phase. Fibroblast activation occurs from tissue injury leading to inflammation and transformation. CAFs are mesenchymal-like cells [3] with an activated fibroblast population phenotype associated with tumor-promoting properties. Tumor-promoting phenotypes mediated by CAFs are invasion and metastasis, ECM remodeling, angiogenesis, metabolic reprogramming, immune suppression, and resistance to radiotherapies, immunotherapies, and chemotherapies [40].

CAFs are amongst the most abundant cell types within the TME of many solid tumor types. For example, activated CAFs account for up to 90% of total PDAC tumor volume [6,9,41]. Activated CAFs are known to support tumor growth and secrete chemokines and cytokines like TGFβ, vascular endothelial growth factor-A (VEGF-A), other angiogenic factors, prostaglandin-2 (PGE2), and indolamine-2,3-dioxygenase (IDO) to promote immunosuppression, where the latter is known to be secreted by inflammatory CAFs [6,9,40,42,43]. Increased TGFβ and VEGF-A secretion by CAFs also increases regulatory T-cell (T-reg) infiltration in adenocarcinomas [43]. TGFβ, PGE2, and IDO are also known to downregulate NCRs and inhibit cytokine secretion from NK cells [44], thus decreasing their cytotoxicity.

CAFs are capable of secreting metabolites [2], which are essential for supporting cancer cell growth once nutrients become scarce and the TME has become hypoxic. CAFs are also responsible for generating desmoplasia, depositing ECM proteins and generating fibrosis in the tumor stroma. This desmoplasia is known to create a physical barrier between cancer cells and therapeutic agents and immune cells, generating interest to target CAFs as a novel therapeutic strategy. However, the multitude of complex functions CAFs perform poses a challenge when developing targeted therapies.

### CAF Heterogeneity and Plasticity

In the past decade, research on CAF function has blossomed to support the development of novel targeted therapies against CAFs to overcome immunosuppression and fibrosis in the TME to enhance delivery of therapeutic agents and/or to increase immune cell infiltration. However, recent discoveries have identified that CAFs exist as a heterogenous population exhibiting both tumor-promoting and tumor-suppressive properties, introducing a challenge when trying to develop targeted therapies against CAFs.

Recent single cell analysis of PDAC tumors demonstrated that the majority of PDAC CAFs are characterized by two distinct subpopulations of activated CAFs, including myofibroblasts (myCAFs) and inflammatory fibroblasts (iCAFs) [18]. myCAFs are characterized by a phenotype of high levels of αSMA which is driven by increased TGFβ expression [16,17]; TGFβ is known to also inhibit production of IFN-γ and TNFα from NK cells [15]. Activated PSCs support an inflammatory response exhibiting low levels of αSMA, but high levels of interleukin (IL)-6 family members, including IL-6 and IL-11, which characterize the phenotype for iCAFs [17,26]. IL-6 is known to influence DC maturation and MDSC differentiation [2,17,40]. Although these data on CAF subpopulations were examined in a PDAC model, the phenotypes between myCAFs and iCAFs have been consistent across different solid tumor types [26,45].

Both myCAFs and iCAFs express variable but notably high levels of FAP, another CAF marker [6,7]. FAP is a member of the prolyl dipeptidyl aminopeptidase (DPP) family and is a transmembrane cell surface serine protease having both endopeptidase and exopeptidase activity, the latter mediated by its DPP activity [46]. FAP cleaves a Pro-X amino acid bond and has collagenase activity [47], which is essential for ECM remodeling since collagen is highly abundant in the stroma and contributes to fibrosis. FAP is involved with processing cytokines and chemokines [48,49,50] in the TME. Interestingly, like myCAFs, FAP expression in CAFs is also induced by TGFβ [51]. FAP is highly expressed in many solid epithelial tumors and is correlated with worse prognosis [43,52], so it has become a novel target of interest in developing cancer therapies. Along those lines, αSMA is also regarded as an important CAF marker since there is a higher abundance of myCAFs compared to other CAF subpopulations [6] but αSMA is not expressed in all CAFs in tumors. To note, CAFs exhibit a wide variety of markers including PDGFRα [3], but FAP and αSMA are the two most highly abundant markers in a variety of solid tumors.

Different CAF subpopulations localize in different compartments of the tumor stroma. myCAF activity is dependent on cancer cell contact so they are located adjacent to cancer cells, especially ones that co-express high levels of αSMA and FAP. Contrarily, iCAFs are localized juxtatumorally where their activity is not dependent on cancer cell contact like myCAFs, displaying how CAF subpopulations exhibit distinct phenotypes [6]. Concurrently, these CAF phenotypes display plasticity during tumor progression. For example, FAP^+^ CAFs in PDAC are also αSMA^−^ during early stages of tumor formation, such as in PanINs (pancreatic intraepithelial neoplasia). As tumors develop, these CAFs become FAP^+^/αSMA^+^ [2]. Although there are other CAF subpopulations present within the TME of solid tumors, myCAFs and iCAFs represent the majority of these cellular subpopulations.

The origin of CAFs may contribute to their plasticity since CAFs can originate primarily from parental tissue, adjacent tissue, mesenchymal stem cells (MSCs), or from bone marrow (BM) [3,9]. However, there is evidence that CAF phenotypes can be reversed to a quiescent state via administration of retinol or vitamin-D [9,43] leading to increased intracellular lipid composition, further demonstrating plasticity. This plasticity allows CAFs to execute their multiple functions. Further studies to understand the function of different CAF subpopulations are critical to distinguish pro-tumorigenic versus anti-tumorigenic CAFs, and therapeutically exploit this cancer-related biology. It is speculated that CAF plasticity may cause challenges in that if one subpopulation is targeted, then another one may compensate its activity for the targeted subpopulation and shift its phenotype to become similar to the targeted CAFs. However, this has yet to be shown in preclinical and clinical investigations but cannot be overlooked.

## 4. Cancer-Associated Fibroblast and Immune Cells in the Tumor Microenvironment

CAFs are known to exhibit crosstalk with immune cells in the TME and influence their phenotypes. For example, CAFs can induce the formation of M2 pro-tumorigenic/anti-inflammatory macrophages, from anti-tumorigenic/pro-inflammatory M1 macrophages, by secreting IL-8 and CXCL12 [40,43]. A positive feedback loop is generated since M2 macrophages go on to further activate more CAFs and stimulate epithelial-to-mesenchymal transition (EMT) in CAFs, especially within FAP^+^ CAFs since FAP^+^ CAFs have a positive correlation with the number of M2 macrophages in the TME [40,43]. CAFs also induce MDSC differentiation, induce neutrophil activation, and inhibit CD8^+^ cytotoxic T-cell proliferation by secreting IL-6 [40,43]. Furthermore, M2 macrophages, T-regs, DCs, and MDSCs all secrete TGFβ to further induce immune suppression in the TME. Interestingly, ethanol is found to increase expression and secretion of TGFβ from stellate cells, which can inhibit NK cell activation [53] suggesting that extrinsic factors influences NK cell activity. MDSCs are known to inhibit NK cells via the membrane bound form of TGFβ and can be recruited by CCL2 secreted from FAP^+^ CAFs [40,43].

IL-6 production by iCAFs suggests iCAFs are involved in immune suppression since IL-6 secreted from DCs inhibits NK cell function [44]. CAFs also secrete TNFα, which causes differentiation and recruitment of helper T cells (Th2), and high Th2 infiltration is correlated with worse prognosis [43]. CAFs can also inhibit NK cell function by secreting various cytokines, chemokines, and MMPs [43].

This brief introduction highlights the complex crosstalk between immune cells and CAFs and that similar molecules inhibit both NK cells and other immune effectors from eliciting their cytotoxic activity. However, important details regarding the crosstalk between CAFs and immune cells, with respect to NK cells, remain poorly understood. Addressing these knowledge gaps should prove to be useful to better exploit NK cell cytotoxicity against cancer cells.

### CAFs and NK Cells in PDAC TME

PDAC is the most common type of pancreatic cancer, with a 5-year survival rate of less than 10%. Poor survival in PDAC is partially attributed to its dense desmoplastic and immune suppressive stroma, mainly composed of the subset of CAFs in the PDAC stroma, called pancreatic stellate cells (PSCs). Experimentally, PDAC tumors grow more rapidly in vivo when co-injected with PSCs [54,55,56], and PSCs increase desmoplasia in the TME. Another study found that injecting PDAC cells with increased TGFβ expression increased desmoplasia, and although this study did not examine PSCs function it suggested that TGFβ is involved in PSC activation [57]. Cytokines produced from fibroblasts and other cells in the TME are known to regulate NK cell activity, where TGFβ can decrease expression of the activating receptor NKG2D on NK cells [53,58], further illustrating the complex crosstalk between PDAC TME and NK cells. Interestingly, IL-6 is known to decrease NK cell-mediated IFN-γ secretion [45], increasing metastatic potential [25,35], further supporting the importance of NK cell function in pancreatic and other solid tumors.

NK cells can target activated PSCs via NKG2D-MICA/B interactions to mediate PSC lysis [58,59,60]. This NK cell-PSC interaction suggests PSCs might divert the attention of immune effectors away from malignant PDAC cells to promote malignant epithelial cell proliferation and survival in PDAC. In PDAC patients, increased NK cell activity has been correlated with better clinical outcomes [17,18,58] and this is also relevant to other cancer types. Despite the efficacy of NK cells in their innate immune response, PDAC still persists. In a recent study, tumor-infiltrating lymphocytes (TILs) were analyzed from both healthy and PDAC patients [58]. Both patient cohorts had high numbers of NK cells, but PDAC patients have low amounts of NK cells as TILs, and NK cell activity was decreased due to a downregulation of CXCR2 [58], a chemokine that is involved in NK cell migration. These data suggest NK cells alone are insufficient to suppress PDAC growth, or that NK cell activity is inhibited during tumor progression, but there is much evidence for the latter.

A single cell RNA-seq analysis study of PSC subpopulations, by Elyada et al. found a novel but smaller PSC subpopulation termed antigen-presenting CAFs (apCAFs). apCAFs may be involved in immunosuppression of CD4^+^ T-cells in PDAC and act as immune “decoys” to prevent tumor cell lysis and may induce T-reg activation [57]. apCAFs present MHC molecules but no subsequent co-stimulatory molecules to stimulate T-cell cytotoxic activity [17,40,57], supporting the idea that CAFs are involved with immune evasion in PDAC. Compared to myCAFs and iCAFS, the abundance of apCAFs in the TME is significantly smaller and little is known about their tumor localization. However, apCAFs are highly plastic since they can shift their phenotypes to be similar to either iCAFs or myCAFs [57].

There has been increasing interest in targeting PSCs to overcome immunosuppression in PDAC; however, the mechanisms of how specific PSC subsets mediate immunosuppression and/or evasion still remains to be explored in depth. Simultaneously, the phenomenon of NK cells mediating lysis of CAFs is not tumor specific and has been observed in a variety of tumor types.

Activated CAFs are known to decrease NK cell cytotoxicity, demonstrated by decreased expression of IFN-γ, perforin, granzyme B, and several activating NK cell receptors using in vitro co-culture experiments of PSCs or colorectal fibroblasts with NK cells [42,61]; further illustrating the complex crosstalk between NK cells and PSCs. Despite the arguments these studies posed, they did not: (1) specify the molecular mechanism of the NK cell-CAF interactions, (2) determine which activated PSC subpopulation was responsible for this phenotype, (3) examine which NK cell subpopulation was most affected, (4) examine changes in NK cell cytotoxicity upon CAF coculture or (5) validate findings using in vivo models. Additionally, these studies did not explore the expression of NK cell ligands on CAFs, which could determine if the expression of NK cell ligands on CAF cell surface molecules mediate NK cell function. Filling these gaps in knowledge may lead to approaches that modulate activated CAFs for the purpose of enhancing cytotoxic targeting of NK cells to malignant cells, a concept that would be relevant to other solid tumors with dense desmoplastic stroma.

## 5. Targeting Cancer-Associated Fibroblasts

Although targeting CAFs and NK cells has not yet been performed simultaneously, the following examples highlight the potential synergy of targeting CAFs and NK cell receptor-ligand interactions and exploiting NK cell activity to improve cancer treatment. Multiple approaches to exploit NK cell function in cancer therapies have been employed, including, but not limited to cytokine therapy, ex vivo NK cell expansion, adoptive transfer therapies from healthy donor NK cells, CAR-NK cells, increasing the potency of ADCC, and generating immunoconjugates [62]. Expansion of NK cells ex vivo has also shown clinical promise as a diagnostic factor for disease-free survival and as a potential treatment modality [58] in clinical investigations.

While there is no single pan-CAF marker, the literature reviewed here provides insights into understanding CAF heterogeneity and provides a rationale to develop CAF subset-targeted stromal therapies as compared to targeting all the CAF subpopulations in tumors, which have detrimental effects on tumor suppression [9]. Along those lines, studies to deplete specific CAFs or attenuate signaling in CAFs have unraveled the subpopulations that are either tumor-promoting or tumor-restrictive. Additionally, as compared to cancer cells, stromal cells have less genetic instability and are less likely to develop resistance to therapies or develop mechanisms of immune escape [8]. The following studies demonstrate the opportunities to target and ablate specific pro-tumorigenic CAF subpopulations to enhance the antitumor immune response and/or increase the efficacy of therapeutic agents in PDAC.

### 5.1. Targeting αSMA^+^ CAFs in PDAC

Özdemir et al. targeted αSMA^+^ PSCs using a PKT (Ptf1a^Cre/+^; LSL-Kras^G12D/+^; Tgfbr2^flox/flox^) genetically engineered mouse model (GEMM). This mouse model is a pancreas-specific PDAC model using a Cre-recombinase Lox-P system driven by the endogenous pancreas transcription factor-1a (Ptf1a) [63] to garner the aforementioned Kras mutation, which is commonly found in human PDAC tumors. The PKT mouse was crossed with an αMSA-tk mouse to generate PKT-αSMA-tk progeny. αSMA^+^ PSCs were depleted following administration of ganciclovir in vivo and found that despite ablating 80% of αSMA^+^ PSCs in the stroma and decreasing fibrosis, αSMA depletion worsened tumor progression and increased resistance to chemotherapy [63]. These αSMA-depleted tumors were poorly differentiated and necrotic, and the findings were also recapitulated in the commonly used KPC (LSL-Kras^G12D/+^, LSL-Trp53^R172H/+^, Pdx-1-Cre) PDAC GEMM [63]. This was the first study and model to identify αSMA^+^ CAFs, primarily myCAFs, as a tumor-restrictive CAF subpopulation.

Patients with lower expression of αSMA within the TME of solid tumors, such as in PDAC, HNSCC, and CRC [63,64], tend to have worse prognosis, further supporting the interpretation that αSMA^+^ CAFs restrict tumor growth. Considering αSMA^+^ PSCs tend to accumulate adjacent to cancer cells, they may provide a structural barrier to limit tumor growth. In line with this concept, αSMA depletion alters the ECM organization in PDAC, indicating that myCAF tumor-restrictive properties are overcome throughout disease progression, despite their high αSMA expression. In the Özdemir et al. study, high levels of FAP^+^ PSCs in the tumor still remained, however the colocalization of αSMA and FAP was diminished, indicative of myCAF depletion, also suggesting that FAP^+^ PSCs are pro-tumorigenic.

αSMA depletion also modulated the immune TME by increasing the number of T-regs and decreasing the number of effector T-cells in the PDAC stroma. Otherwise, there was minimal examination of NK cell function except that their numbers did not change upon αSMA^+^ PSC depletion. This study provided no information regarding PSC maturation states or activation. Interestingly, myCAFs in liver metastasis from an in vivo pancreatic cancer model promoted angiogenesis [65], further indicating that each CAF subpopulation has a complex set of functions. In addition, αSMA^+^ CAFs cannot be easily targeted clinically, since it is not expressed on the cell surface, but this study provided important insights regarding myCAF function in PDAC.

### 5.2. Targeting Shh Signaling in CAFs in PDAC and Other Malignancies

Sonic Hedgehog signaling is overexpressed by PSCs in PDAC and also contributes to pancreatic desmoplasia. Therefore, Rhim et al. generated a transgenic mouse model called ShhPKCY (Shh^flox/flox^; p53^fl/+^; Kras^LSL-G12D/+^; Pdx1-Cre; Rosa26^LSL-YFP^) to perform targeted depletion of Shh in the pancreas and to visualize epithelial cells during tumor progression via YFP expression [66,67]. Rhim et al. found similar results as Özdemir et al. did with their αSMA depletion. Shh depletion caused tumors to become aggressive and increased metastasis. These results were also validated in the well-known KPC PDAC model when mice were treated with a Smoothened (Smo) inhibitor, where Smo is released upon Shh binding to the Patched1 (Ptch1) receptor [68]. Further analysis indicated strong Gli expression in αSMA^+^ CAFs, suggesting that myCAFs were affected, further identifying myCAFs as a tumor-restrictive subpopulation. However, there were residual CAFs with low αSMA expression that were also positive for Gli expression, where Gli is a downstream effector of Shh signaling. Interestingly, both of these studies saw an increase in EMT markers, which is typically associated with an aggressive phenotype in cancers, like metastasis. Similarly, Liu et al. used a GEMM that had specific genetic depletion of Smo in fibroblasts but maintained a Kras mutation in the epithelium (Mist^KrasG12D/+^; Fsp-Cre; Smo^LoxP/−^) [68]. From this in vivo model, they found that Shh depletion in PSCs increased ductal metaplasia by causing non-canonical activation of the transcription factor Gli2, which increased expression of TGFα to induce activation of the proliferative pathway molecules EGFR and Akt [68]. Although these studies did not examine immune infiltration upon Shh depletion, patient data in gastric cancer correlated increased Hedgehog signaling and decreased NK cell infiltration within the tumor stroma [69], in accordance with the correlation of low NK cell infiltration with poor overall survival in these patients.

### 5.3. Targeting NetG1^+^ CAFs in PDAC

A recent study identified NetrinG1 (NetG1) as a relevant and novel CAF target in PDAC with an inverse correlation of NetG1 expression and patient survival [70]. Francescone et al. found that targeting NetG1-expressing CAFs with a neutralizing monoclonal antibody decreased NK cell inhibition and increased their infiltration in PDAC models via upregulation of IL-15 and downregulation of IL-6 [70]. Targeting NetG1 did not change the abundance of αSMA^+^ myCAFs within the PDAC TME [70]; therefore, it can be hypothesized that NK cells may localize with IL-6 expressing iCAFs rather than myCAFs to inhibit NK cell infiltration.

### 5.4. Targeting FAP^+^ CAFs in PDAC

Feig et al. demonstrated that FAP^+^ PSC depletion in PDAC allowed for increased response to ICIs, to which PDAC is generally unresponsive to [60]. They generated a GEMM PDAC model using KPC mice and introduced a bacterial artificial chromosome transgene for a modified FAP gene that drives diphtheria toxin (DT) receptor expression so upon DT administration FAP^+^ PSCs were depleted [60]. FAP^+^ PSC depletion increased tumor susceptibility to anti-CTLA-4 and anti-PD-1/anti-PD-L1 immunotherapies, the latter having a greater effect. There was also a decrease in CXCL12, a chemokine ligand expressed by FAP^+^ CAFs that binds to the CXCR4 receptor on cancer cells, diverting T-cells from malignant cancer cells. Interestingly, NK cells migrate to cells that secrete CXCL12 [16], so NK cells may be diverted from PDAC cells to FAP^+^ CAFs via this mechanism, supporting the hypothesis that FAP^+^ CAFs may mediate immune evasion.

Recently, a novel immunocytokine, FAP-IL-2v (RO6874281), has been and is continued to be used in clinical trials for PDAC and other cancers [71,72]. RO6874281 targets FAP^+^ CAFs and stimulates NK cell activation via an IL-2 variant, demonstrating interest to target NK cell-CAF interactions, therapeutically [71,72].

## 6. Other Approaches to Exploiting NK Cell Function in PDAC

Using a combination of a Cdk4/6 inhibitor with a MEK inhibitor increased NK cell activity in a KP model of lung adenocarcinoma (LA) and in LA cell lines from a KPC PDAC mouse cell line [73]. This combination therapy also induced expression of the NK cell ligands MICA and ULBP2, further underscoring the importance of innate immunity in Kras driven cancers, such as PDAC. Despite an increase in CD4^+^ and CD8^+^ T-cells, there was no increase in T-cell activity, nor were there changes in macrophage or B-cell populations following this combination therapy [73]. Although Kras is mutated and constitutively active in these models, MEK inhibitors can inhibit MEK signaling and subsequent secretion of IL-6 and other cytokines that inhibit NK cell function and promote cancer cell growth. However, the rationale to use it for NK cell expansion is problematic, since MEK signaling is necessary for the production of proteins necessary for NK cell cytotoxicity.

Although not a direct exploitation of NK cell activity, it was observed that gemcitabine treatment following PDAC resection caused a decrease in MDSC populations, and increased NK cell activity and numbers associated with the efficacy of adjuvant chemotherapy [74,75]. This finding demonstrates the importance of the innate immune response in PDAC and was relevant because these in vivo experiments recapitulated findings from clinical trial(s), since gemcitabine is a widely used PDAC therapeutic agent.

In other preclinical PDAC models, Lee et al. took an interesting and novel approach to increase NK cell tumor infiltration. They generated a NK cell-recruiting protein-conjugated antibody (NRP-body) using a Meso-scFv-Fc-CXCL16 construct. This NRP-body selectively targets mesothelin expressing cancer cells and has a furin cleavage site to release of CXCL16 from the NRP-body into the PDAC TME [62]. Mesothelin is an appropriate target since it is overexpressed in PDAC, which also expresses furin to allow cleavage of the NRP-body. CXCL16 is a chemokine that recruits NK cells; therefore, increased CXCL16 in the TME increased NK cell infiltration in PDAC murine tumors. The NRP-body was used in combination with ex vivo expanded NK cells and caused decreased tumor progression and increased overall survival in solid and metastatic tumor models [62]. The expanded NK cells were also site-specific and, in their metastasis model, were localized in the lungs and liver, which are common metastatic sites in PDAC. Due to the NRP-body’s selectivity, there were no reported toxicities. CXCL16 binds to NK cells via the CXCR6 receptor, causing increased Erk activity in NK cells, and subsequently increased granulation and cytokine production. Although no data were shown, CXCL16 is known to increase CD8^+^ T-cell function, so there may be T-cell mediated cytotoxicity within the TME [62]. However, a finding that seems discordant to NK cell activity is that CXCL16 caused an increased ratio of CD56^bright^ versus CD56^dim^ NK cells [62], the latter being more cytotoxic. Concurrently, there was decreased tumor progression, but this effect needs to be further characterized. Moreover, the study focused on the expression of chemokine receptors on ex vivo expanded NK cells and not on naïve NK cells, which may not be beneficial since not all patients can tolerate nor opt for ex vivo NK cell expansion. However, many other enzymatic targets can be manipulated in this NRP-body construct, such as targeting FAP-expressing cells in PDAC since FAP has enzymatic activity and is overexpressed in both PDAC CAFs and cancer cells.

Lo et al. developed and explored the potency of FAP-CAR-T cells in cancers with low to moderate immunogenicity, including lung and pancreas, and performed a combination study with the chemotherapy agent gemcitabine in a PDAC experiment, suggesting clinical promise for targeting CAFs in a combination chemotherapy setting [48,49,76]. The FAP-CAR-T cells also depleted approximately 70% of αSMA^+^ CAFs and increased CD8^+^ T-cell infiltration into tumors. FAP is known to mediate fibrosis and vascularization, and FAP-CAR-T cells decreased both of these properties [48,49,74]. This FAP-CAR-T construct did not cause bone toxicity or cachexia, indicating there was higher specificity for a CAF-restricted FAP antigen epitope in tumors [48,49]. The difference in these CAR-T constructs may be related to the FAP epitope being targeted since FAP in stromal cells may present a different antigen than in BMSCs. Therefore, it is critical to understand the antigen of interest when designing CAR-T cells for targeted therapies [48,49,76,77].

Hyaluronan (HA), an abundant component of the PDAC TME, is enriched in tumors with low NK cell activity and cancers with high expression of HA and Proteoglycan Link Protein-3 (HAPLN3) have poorer prognosis [18]. This phenomenon is interesting because targeting FAP is known to decrease HA, which can improve NK cell cytotoxicity to malignant cells, but this has yet to be proven. Although Lo et al.’s study did not explore the role of hypoxia on NK cell function, this demonstrates that targeting FAP^+^ CAFs has therapeutic potential in more than one tumor type. Interestingly, FAP^+^ PSCs may play a role in suppressing T-cell activity [60], hence making them an ideal target to increase the anti-tumor immune response.

Cytokine therapies, including IL-2 and IL-15, are known to enhance NK cell cytotoxicity in preclinical models and are clinically employed to enhance NK cell function in tumors with high numbers of NK cells present. IL-2 is a cytokine found in T-cells that binds to IL-2 and IL-15 receptors on cytotoxic lymphocytes, such as NK cells, but IL-2 can also stimulate production of immunosuppressive T-regs [78]. IL-15 is a cytokine that increases expansion of NK cells and CD8^+^ T-cells, but not T-regs [78]. IL-15 can increase the expression of NCRs in DCs [44], and continues to be evaluated in clinical trials. IL-2 can also cause severe toxicities; therefore, IL-15 may be more efficient and is generally preferred, but all cytokine therapies can have systemic toxicities [16].

In many in vitro and in vivo studies, IL-2 and IL-15 have been utilized to enhance NK cell-mediated lysis of cancer cells. Interestingly, Van Audenaerde et al. used IL-15 in vitro to stimulate NK cells and observed lysis of PDAC cells and PSCs by upregulating the NKG2D receptor [79] demonstrating that CAFs can be targets of stimulated NK cells.

## 7. Conclusions

**Figure 1:**
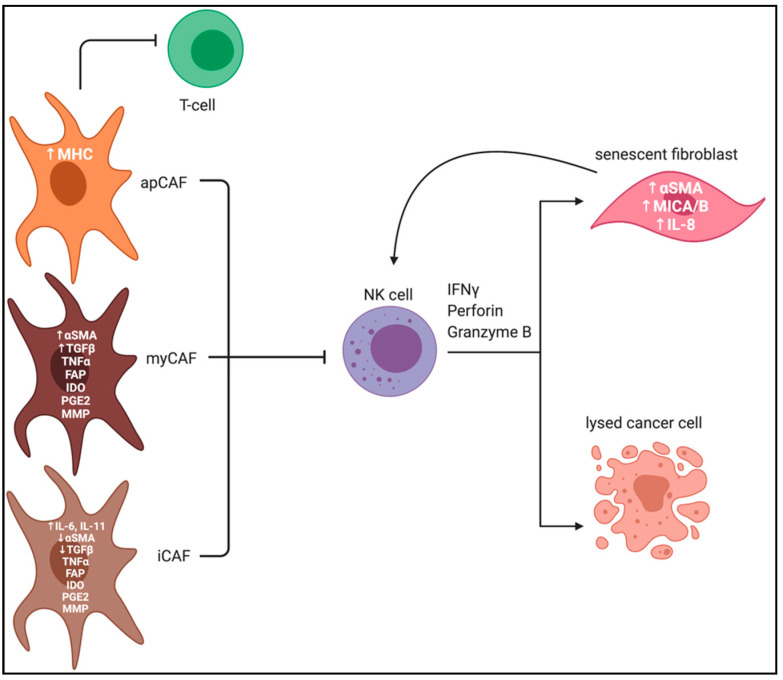
Cancer-associated fibroblast subpopulations influence NK cell activity. Activated CAFs, including myCAFs, iCAFs, and apCAFs, secrete a variety of cytokines, chemokines and MMPs to inhibit immune cell activity, particularly inhibiting NK cell cytotoxicity against malignant epithelial cells. CAF subpopulations have variable expression of distinct markers, including αSMA, IL-6 and FAP, the latter potentially targetable to overcome CAF-mediated NK cell immunosuppression. Senescent fibroblasts [80] secrete IL-8 to stimulate NK cell activity to allow for NK-cell mediated lysis of fibroblasts [Created with BioRender.com].

We have described efforts to further understand the functions and relationships between CAFs and NK cells within the TME and how to target them in multiple tumor types. Fibroblasts and CAFs are heterogenous and exhibit multiple functions to influence processes within the TME, and now known to be tumor-promoting and tumor-restrictive. Figure 1 summarizes the known influences of fibroblast subpopulations on NK cell activity, where both myCAFs and iCAFs can secrete many of the same cytokines to inhibit NK cell function despite exhibiting distinct phenotypes. However, while NK cells crosstalk with CAFs to influence their activity, this crosstalk is still poorly understudied.

Although there are no targetable markers for iCAFs, it would be interesting to examine their depletion on NK cell function to further characterize this subpopulation. However, targeting FAP shows the most promise since it is expressed on the surface of many CAFs subpopulations, leading to potential potent cytotoxic events in tumors and a way to bypass immune suppressive mechanisms outlined by CAFs. However, there is evidence that NK cells may mediate immune selection pressure on both cancer cells and CAFs, which may attribute to increasing or decreasing expression of NK cell ligands to induce immune evasion of NK cells from malignant epithelial cells. This is a hypothesis that still needs to be tested in tumors with high NK cell abundance.

CAFs can mediate NK cell inhibition by engaging inhibitory NK cell ligands, but it is unclear if CAFs cause NK cells to shift to an immature phenotype to decrease their function or exhaust them. If the latter, therapies to enhance NK cell function may have modest but still potent effects. It would also be interesting to determine if NK cells preferentially lyse a particular CAF subpopulation, and examine if it correlates with CAF subpopulation localization within the TME. Additionally, examining expression levels of NK ligands by CAF subpopulations, and determining if CAFs influence the receptors expressed on NK cells, would lead to better characterization of the molecular mechanisms driving NK cell-CAF interactions. Accordingly, NK cell heterogeneity will also create a challenge of teasing apart these various possibilities without additional studies. Although there is no single marker for CAFs, targeting an activated subpopulation like FAP^+^ CAFs may render cancer cells susceptible to immune invasion and to diverse immunotherapies.

Overall, it is abundantly evident that NK cell content correlates with minimal stromal content in the TME, with increased cancer survival demonstrated in preclinical and clinical settings. This review has described many novel and creative methods used to target CAFs and investigate NK cell function in the PDAC TME. The mechanisms facilitating NK cell-CAF interactions and crosstalk still remain unknown but creates opportunities for important new research.

## Figures and Tables

**Table 1:** Known activating and inhibitory receptors and ligands in NK cells in mice and humans [9,10,11,13,14,23,25,26,27,28,29,30,31,32]. KIRs have a particular nomenclature: the first number following KIR indicates the number of immunoglobulin (Ig)-domains on the extracellular portion of the receptor, the second number indicates the length of the cytoplasmic tail and its activation state [19]. For example, KIR2DL4 has two Ig-domains (2D) and the length of its cytoplasmic tail represented by L or S where, they are either inhibitory or activating, respectively. TIGIT and DNAM-1 are other NK cell receptors that are also present on other immune cells, like T-cells.

NK Cell Receptor Class/Family	Receptor (Human/Mouse)	Ligand(s) (Human/Mouse)	Activating or Inhibitory
NCR	NKG2D/*mNKG2D*	MICA/B, ULBPs/*Rae-1, H-60, MULT1c*	Activating
	NKG2C/*mNKG2C*	HLA-E/*Qa1b*	
	NKp30 (human only)	Heparin, HLA-B3 (BAT3), B7H6	
	NKp46/*Ncr1*	Heparin/*Heparin*	
	NKp65 (human only)	CLEC2A (skin specific)	
	NKp80 (human only)	AICL	
	NKp44 (human only)	Heparin, NKp44L	
		PCNA	Inhibitory
	CD94-NKG2A/*mCD94-NKG2A*	HLA-E/*Qa1b*	Inhibitory
KIR	KIR2DS1/2	HLA-C1/2	Activating
	KIR2DS4	HLA-A	
	KIR3DS1	HLA-B	
	KIR2DL1–3	HLA-C	Inhibitory
	KIR2DL4	HLA-G	
	KIR3DL1	HLA-B	
	KIR3DL2	HLA-A	
Ly49 (mouse only)	*Ly49D*	*H-2D*	Activating
	*Ly49H*	*M157*	
	*Ly49A*	*H-2D*	Inhibitory
	*Ly49C*	*H-2D*	
	*Ly49I*	*H-2k*	
	*Ly49P*	*H-2D*	
Ig Superfamily	DNAM-1 (CD226)/*mDNAM-1*	CD155, CD122/*CD125, CD112*	Activating
Other NK cell receptors	TIGIT/*mTIGIT*	CD155, CD122/*CD125, CD112*	Inhibitory

## Data Availability

No new data were created or analyzed in this study. Data sharing is not applicable to this article.
